# The effectiveness of a dedicated central venous access care team to prevent catheter-related bloodstream infections

**DOI:** 10.1017/ash.2022.114

**Published:** 2022-05-16

**Authors:** Fortune Charles Fil de Lara, Maria Jesusa Mano, Karl Evans Henson, Jia An Bello, Cybele Lara Abad

## Abstract

**Background:** Catheter-related bloodstream infection (CRBSI) rates remain high despite the use of an insertion bundle. We hypothesized that line care and maintenance by a dedicated team would help decrease CRBSI rates. This study was conducted in The Medical City (TMC), is a 526-bed, private, tertiary-care center in Pasig City, Philippines. **Methods:** All adult hospitalized patients from October 1, 2020, to October 31, 2021, with a newly inserted temporary central venous catheter (CVC) were eligible for inclusion. CRBSI rates before the intervention (October 2019 to March 2020) and after the intervention (April to October 2021) were compared. The intervention arm consisted of a dedicated central venous access team (CVAT) who provided education and performed daily line care and dressing changes per protocol. A series of χ^2^ and Wilcoxon rank-sum tests were performed to compare characteristics between exposure groups. Incidence rates of CRBSI before and after the intervention were compared using an incidence rate ratio approach. **Results:** In total, 209 CVCs were enrolled in the study, with 103 CVCs (49.28%) in the preintervention arm and 106 CVCs (50.72%) in the postintervention arm. Baseline patient characteristics were similar. CRBSIs were more frequent in the preintervention arm than the postintervention arm (39 of 103 vs 28 of 106; *P* = .08). The CRBSI incidence density rate was higher in the preintervention arm than the postintervention arm, but the difference was not statistically significant (37.46 per 1,000 patient days vs 25.97 per 1,000 patient days; *P* = .14). Median time to CRBSI was similar in both groups (9 vs 8 days). **Conclusions:** Baseline CRBSI rates were high and risk of infection increased by day 8 after line insertion. We detected a decreasing trend in rates of CRBSI with a dedicated CVAT, but multiple interventions are likely needed to influence overall rates.

**Funding:** None

**Disclosures:** None

